# Is the Prevalence of Low Physical Activity among Teachers Associated with Depression, Anxiety, and Stress?

**DOI:** 10.3390/ijerph19148868

**Published:** 2022-07-21

**Authors:** Elżbieta Biernat, Monika Piątkowska, Michał Rozpara

**Affiliations:** 1 Collegium of World Economy, SGH Warsaw School of Economics, Al. Niepodległości 162, 02-554 Warsaw, Poland; 2 Faculty of Physical Education, Józef Piłsudski University of Physical Education in Warsaw, 34 Marymoncka, 00-968 Warsaw, Poland; monika.piatkowska@awf.edu.pl; 3 Institute of Sport Sciences, The Jerzy Kukuczka Academy of Physical Education, 72A Mikołowska, 40-065 Katowice, Poland; m.rozpara@awf.katowice.pl

**Keywords:** mental health, education, physical exercise

## Abstract

The aim of this study was to analyze the levels of stress, depression, and anxiety among Polish secondary school teachers and their association with selected psychosocial, sociodemographic, and lifestyle factors. A cross-sectional study was conducted among 116 Polish teachers in 2019, using the International Physical Activity Questionnaire—Short Form (IPAQ-SF), the Depression and Anxiety Stress Scale (DASS-21), and author questionnaire including psychosocial factors specific to the respondents’ work environment. The prevalence of moderately to extremely severe levels of depression, anxiety, and symptoms of stress in teachers was 18.1%, 22.4%, and 51.7%, respectively. Among respondents with moderate or high physical activity level (PAL), normal or mild levels of depression (OR = 3.62; CI [1.31–10.03]), anxiety (OR = 2.61; CI [1.01–6.73]), and stress (OR = 2.79; CI [1.16–6.69]) were more common. The level of individual symptoms of mental disorders was higher than indicated by previous Polish reports. Given that teachers’ low PAL is significantly related to their moderately to extremely severe levels of stress, depression, and anxiety, we suggest running leisure-time physical activity (LTPA) enhancement programs and supporting the leadership of school management and the higher-education system in this regard.

## 1. Introduction

Teachers—both in Poland [1] and internationally [2]—represent one of the largest professional groups. At the same time, they have the highest dropout rate among those working in the public sector [3,4]. Their working environment is full of stressful factors [4,5]. These are physical (e.g., high noise levels, artificial lighting) and psychosocial (e.g., inappropriate student behavior, overcrowding in schools, low status of the profession) [3]. Whereas the former factors have a direct impact on health and can be accurately measured, the latter do not translate directly into health risks. However, depending on the importance of a given factor for the individual and his/her emotional response, they may lead to changes in the body that will result in loss of health [3,6]. The most commonly confirmed health consequences of occupational stress include coronary heart disease [7,8,9,10], alcoholism, and psychiatric disorders [11]. Stress may correlate with the risk of metabolic syndrome [12], acute myocardial infarction [13], type 2 diabetes [14,15], and renal dysfunction [16].

The teaching profession has been described as one of the most stressful and depressing [17,18]. In Germany, symptoms of significant mental health problems have been reported in almost 30% of teachers [4], and in Malaysia in more than 20% of those working in secondary schools [2]. This problem is also reflected in the Polish reality. As many as 86% of teachers believe that their workload is higher than in other professions [19], 20% have full symptoms of burnout syndrome [20], 16–17% a high level of organizational burden resulting from conflict situations at work, and 25% a low sense of meaning in their work [21]. It is evident that psychosocial burdens are very strongly related to the psychophysical condition of teachers (e.g., anxiety, depression, or professional burnout) [22,23,24]. Single factors, such as stress [25,26,27], depression [28], and anxiety [29], affect health and teacher effectiveness [30].

Interest in the diagnosis of teachers’ psychosocial burdens and effects on their health (including mental health problems) has an important empirical basis. However, relatively little is known about the relationship of single factors, such as stress, depression, and anxiety, and the risk factors of teachers’ lifestyle (e.g., prolonged sitting, lack of or too little physical activity). Moreover, these types of behaviors can, in the long run, be a direct cause of mental health problems or increase the risk of developing such conditions.

Therefore, the aim of this study was to analyze the levels of stress, depression, and anxiety among Polish secondary school teachers. This research also focused on determining the association of moderate, severe, or extremely severe symptoms of these disorders with selected factors: psychosocial (work experience, total lessons per week, steady income), sociodemographic (gender), and lifestyle characteristics of the respondents (level of physical activity, sedentary behavior, and biometric characteristics). To the best of our knowledge, this is the first study of its kind in Poland. However, as far as lifestyle variables are concerned, only the physical activity results are presented in this study. Sedentary behaviors and biometric traits will be presented in further studies.

## 2. Material and Methods

### 2.1. Study Design and Participants

The study was conducted in June 2019. The sampling frame was a list of all general secondary schools in Katowice, from which eight institutions were selected by simple random sampling. We assumed that the sampling frame was all teachers of high schools in Katowice (*n* = 789) as of 30 September 2018. We estimated the number of teachers based on Wayne’s formula [31]. Interviews with randomly appointed teachers at schools were conducted in the teachers’ workplace in June 2019, with the prior consent of the head teacher. The interview lasted 30 min and was conducted by a trained interviewer. A total of 116 teachers took part in the survey: 28 male and 88 female (humanities—55.2%, mathematics and natural sciences—31.9%, physical education—12.9%). The mean age of the respondents was 45.0 ± 8.8 years. Most teachers (74.1%) were employed full-time. The Bioethics Committee of the Jerzy Kukuczka Academy of Physical Education in Katowice approved the study.

### 2.2. Measurements (Study Instruments and Data Analysis)

The Polish version of the 21-item Depression Anxiety Stress Scale (DASS-21) [32], the Polish short version of the International Physical Activity Questionnaire (IPAQ-SF) [33], and author questionnaire containing questions on gender and psychosocial factors characteristic of the respondents’ work environment (work experience, total lessons per week, steady income) were used (Table 1).

### 2.3. Dependent Variables

The culturally adapted and validated short-version DASS-21 was used to assess the frequency and severity of depression, anxiety, and stress. The internal consistency of the Polish version of the DASS-21 has Cronbach’s alpha values of 0.86, 0.84, and 0.85 for depression, anxiety, and stress subscales, respectively [32]. Respondents were asked to indicate the presence of the symptoms over the past week on a four-point Likert scale, ranging from 0 (did not apply at all) to 3 (applied very much or most of the time). The total score for each subscale was calculated and the severity rating was classified as normal, mild, moderate, or severe to extremely severe [34] (Table 2).

### 2.4. Independent Variables

The IPAQ-SF was used to provide information about the frequency, duration, and intensity of physical activity during the last 7 days. Physical activity refers to every physical effort lasting for at least 10 min at a time [35]. A weekly energy expenditure of total physical activity (EETPA) was calculated by multiplying a MET number attributed to it (vigorous (VPA)—8.0 METs, moderate (MPA)—4.0 METs, walking—3.3 METs) by the number of days of practicing it per week and duration in minutes per day. Subjects were ranked in two score categories concerning physical activity level (PAL): low and moderate/high [36] (Table 1).

### 2.5. Confounding Variables

Gender (female, male), work experience (≤20 years, >20 years), total lessons per week (≤18 h/week, >18 h/week), and steady income (≤2700 PLN, >2700 PLN) were used as confounding variables.

All variables included in the analysis and their respective descriptive statistics are presented in Table 1.

### 2.6. Statistical Analysis

The data collected in the questionnaires were analyzed using SPSS Statistics 26 (IBM Corporation, Armonk, NY, USA). Descriptive statistical analysis was conducted using means (M), standard deviations (SD), median (Me), first and third quartiles (Q1, Q3), frequency (f), and relative frequency (rf) in percentages. The assessment of relationships between depression, anxiety, and stress level (dependent variables) and the level of physical activity (independent variable), as well as gender and psychosocial factors (confounding variables) was based on odds ratios (ORs) and confidence intervals for ORs.

The study used regression modeling based on the technique of sequentially entering the independent and confounding variables of the model, commonly used in physical activity studies [37,38]. The area under the curve (AUC) calculated for the receiver-operating characteristic (ROC) curve was used to assess the predictive value of the models considered (classifier validity assessment) [39]. AUC values were judged based on published standards: AUC ≥ 0.90 outstanding prediction, 0.80 ≤ AUC < 0.90 excellent prediction, 0.70 ≤ AUC < 0.80 acceptable prediction, 0.50 < AUC < 0.70 poor prediction, AUC = 0.50 no prediction [40]. The level of statistical significance was set at α = 0.05.

## 3. Results

The prevalence of moderately to extremely severe levels of depression, anxiety, and symptoms of stress of teachers was 18.1%, 22.4%, and 51.7%, respectively (Table 1). The subjects’ averages for depression and anxiety were at a normal level (M = 7.7 ± 6.8 pts.; M = 6.5 ± 6.2 pts.) and the average for stress was at a moderate level (M = 19.3 ± 7.4 pts.). Descriptive statistics for the DASS-21 subscales are presented in Table 3.

Mean values of energy expenditure of VPA, MPA, and TPA were 602.0 ± 793.2, 502.2 ± 518.2, and 1238.7 ± 976.4 MET-min/week, respectively (Table 3). According to the study, a third of the respondents (32.8%) had a low level of physical activity, 61.2% moderate, and 6.0% high.

Table 4 shows the results of modeling the probability of normal or mild vs moderate to severe depression. Due to the larger number of predictors and fair classification efficiency (AUC = 0.70), we discuss in detail the model_5_ based on the predictors PAL, gender, work experience, total lessons per week, and steady income. Depression was found to be statistically significantly associated with PAL. Teachers who declared moderate or high PAL were 3.6 times more likely to report normal or mild levels of depression than those with low PAL (OR = 3.62; CI [1.31–10.03]). The odds ratio (OR) of reporting a normal or mild level of depression was 43% higher for females than for males, 66% higher for those with ≤20 years of work experience than those with more experience, 46% higher for those working ≤18 h/week than >18 h/week, and 23% lower for those with lower steady income (≤2700 PLN) than the others (Table 4). No statistically significant differences were found in this regard.

As far as modeling the probability of normal or mild vs moderate to severe anxiety is concerned, a model (model_5_) based on the predictors PAL, gender, work experience, total lessons per week, and steady income achieved a classification efficiency of AUC = 0.68 (Table 5). Again, the only statistically significant factor was PAL. Teachers who declared moderate or high PAL were 2.6 times more likely to report normal or mild level of anxiety than those with low PAL (OR = 2.61; CI [1.01–6.73]). The odds ratio (OR) of reporting normal or mild level of anxiety was 44% lower for females than for males and 34% lower for teachers with ≤20 years of work experience than those with more experience. Teachers working ≤18 h of lessons/week and earning ≤2700 PLN were, respectively, 2.7 times and 1.8 times more likely to have normal or mild symptoms of anxiety than those working and earning more.

The model_5_ probability of normal or mild vs. moderate to severe stress had a classification efficiency of AUC = 0.70 (Table 6). Stress was found to be statistically significantly associated with PAL. Teachers who declared moderate or high PAL were 2.8 times more likely to report a normal or mild level of stress than those with low PAL (OR = 2.79; CI [1.16–6.69]). The odds ratio (OR) of reporting normal or mild levels of stress was 65% lower for females than for males and 47% lower for teachers with ≤20 years of work experience than for those with more experience. Teachers working ≤18 h of lessons/week and earning ≤2700 PLN were, respectively, 1.6 times and 2.2 times more likely to have normal or mild symptoms of stress than those working and earning more.

## 4. Discussion

This study aimed to analyze the levels of stress, depression, and anxiety among secondary school teachers in Katowice and to determine the association of moderate, severe, or extremely severe symptoms of these disorders with selected factors: psychosocial work environment, sociodemographic, and lifestyle of the subjects. We can distinguish the following psychological factors: work experience (≤20 years–>20 years), total lessons per week (≤18 h/week–>18 h/week), steady income (≤2700 PLN–>2700 PLN), lifestyle factors (level of physical activity: high or moderate–low), and sociodemographic factors (gender).

Previous research shows that the former may have an impact on teacher frustration [26,41]. Negative emotions are accompanied by pathological biochemical and physiological changes (acceleration of heart rate, secretion of adrenocorticotropic hormones, increase in blood pressure), which consequently lead to loss of health [3,6,42]. Sociodemographic factors (e.g., gender) may condition teachers’ mental state [43,44], and unhealthy lifestyle behaviors may mediate the correlation of occupational stress and chronic disease risk [2,45,46]. The strongest relationships are those in which factors cluster together, reinforcing the interaction [2,47,48]. Therefore, in this study, sociodemographic, psychosocial, and lifestyle variables of teachers were analyzed. To the best of our knowledge, this is the first study of its kind in Poland and one of two in the world [49].

The results of our study firstly show that the prevalence of moderately to extremely severe levels of depression, anxiety, and symptoms of stress of teachers is 18.1%, 22.4%, and 51.7%, respectively. This is far more than reported by Polish population data and previous studies of teachers. According to the Central Statistical Office [50], depressive symptoms of varying severity affect 16.1% of adult Poles (mild—11.9%, moderate—2.7%, severe—1.5%). On average, every sixth person suffers from anxiety disorders [51] (for anxiety attacks ever in life—17.3%, for generalized anxiety—23.9%, for social anxiety—14.9%) [52]. Stress is a problem for 45% of Poles (in 26% of cases due to work) [53]. However, among 14 groups of employees representing social service professions, the highest perceived level of stress is reported in teachers [54]. Every second Polish junior high school teacher experiences it at work often or even always [55], and 43.3% of the teaching staff describe their stress level as high [55]. In the group of high school teachers, the greatest stressor is mental strain, lack of rewards, organizational uncertainty, a sense of threat, and physical nuisance [56]. Our analysis shows that the problem of mental ill health in this professional group is becoming worse. Researchers from other countries also draw attention to the problem of the poor psychophysical condition of teachers. Australians recommend a paradigm shift from burnout to occupational depression, due to the fact that depression coincides with burnout [57]. The Spanish found that many individual, school, and workplace factors can play a large role in altering the state of depression [58]. Among Egyptian teachers, work stress, anxiety, and depression are significantly higher in those over the age of 40 years, female teachers, elementary school teachers, and those with insufficient salaries, higher teaching experience, higher qualifications, and higher workload [59]. However, they did not investigate the level of physical activity of teachers.

The present study demonstrates that among the factors analyzed (gender, work experience, total lessons per week, steady income, PAL), only PAL had a statistically significant (OR = 2.79, [1.16–6.69]) relationship with moderately to extremely severe levels of teacher stress, depression, and anxiety. Teachers who reported moderate to high PAL were 3.6 times more likely (than those with low levels) to report normal to mild levels of depression (OR = 3.62; CI [1.31–10.03]), 2.6 times more likely to report normal to mild levels of anxiety (OR = 2.61; CI [1.01–6.73]), and 2.8 times more likely to report normal to mild levels of stress (OR = 2.79; CI [1.16–6.69]). There were no statistically significant correlations between psychological status and gender, length of service, or gross weekly work hours among the teachers surveyed. This indicates the importance of PA interventions for maintaining mental health in this occupational group, which supports an earlier review analysis by Bogaert et al. [49]. According to that, undertaking PA has a positive impact on perceived job satisfaction and job stress (across various other jobs) [49]. It is well known that PA is a significant source of personal and social capital development, with particular reference to the effects seen in the labor market and the associated economic effects (i.e., employability in later stages of working life or health-care costs) [60]. Regular PA is known to be an enabler for building social resources—support and social inclusion [61,62]. Creating a better social network, social activation, and improved self-esteem can prevent stress or lead to greater stress tolerance [61,63,64]. PA can also mitigate the effects of stress by influencing an individual’s eating habits, stabilizing hormone production, and lowering blood pressure [63,65]. It can affect mental health by moderating mood and emotions, improving self-esteem and cognitive functioning, and counteracting anxiety, stress, depression, aging, and mental dysfunction [66]. A significant association between low PA levels and depression (OR = 2.16, [1.12–4.16]), stress (OR = 0.45, [0.23–0.88]), and personal income (OR = 0.63, [0.44–0.91]) was confirmed in Iranian women [45]. PA of Iranian female high school students was also shown to be inversely related to anxiety [67]. Similarly, Cheung et al. demonstrated that lack of exercise among Hong Kong nursing students was a significant correlate of depression, anxiety, and stress (*p* < 0.05, OR = 0.4–1.6) [68]. A Korean study [69] found that the risk of unhealthy lifestyle factors (including low PA) was more common among those with mild to moderate stress than among those with lower stress levels. Of course, some studies show weak or inconsistent correlations between work stress and unhealthy behaviors [46,70], indicating the need for further inquiry. Finally, there are studies noting that the strength of impact on mental health may depend on the type of PA (active commuting or physical activity at work, at home, in leisure) [71,72]. In the case of Flemish secondary school teachers [49], as well as several other studies involving adults [71,73,74,75], higher participation in leisure-time PA (LTPA) was associated with more positive perceived health. Holtermann et al. speculated that LTPA may be more beneficial due to its dynamic use of large muscle groups and sufficient time to recover from exercise [76]. It may also induce a greater sense of autonomy and mastery [70], which may support intrinsic motivation [77], result in higher self-esteem, and indirectly enhance social cohesion and active coping [64].

All of this confirms the need for a rapid response in the teaching environment and the introduction of strategies to increase PA, which, as previous research has shown, has the potential to combat mental disorders [78]. Mere mental health promotion limited to public education [79], or even providing teachers with methods to cope with stress and tension at work, is not enough, especially since, unlike physical or metabolic diseases, symptoms of depression, anxiety, and stress are often ignored and treated as a normal psychological reaction that does not require treatment [80]. It is obvious and natural that stressors are immanent to the teaching profession [81]. Meanwhile, untreated early disorders can lead to unexplained somatic symptoms [82].

Undoubtedly, further research (of broader scope) is needed to confirm our findings, as well as more detailed analyses (e.g., correlation matrix or multivariate regression analysis) to understand the causal relationships of PA and moderately to extremely severe levels of stress, depression, and anxiety. The data from these studies will be a useful guide for making decisions about improving the mental health and well-being of teachers (as well as related professional groups). Knowing that mental and physical health are significantly interrelated, we can assume that improving one element of teacher health may indirectly improve other elements.

## 5. Conclusions

Teachers of secondary schools in Katowice perceive their mental health to be worse than the general health level of the Polish population. Moreover, the level of individual symptoms of mental disorders is higher than indicated by previous Polish reports on this professional group. This can be a major problem for this environment, as well as for society as a whole, resulting not only in chronic disease [4,17] and reducing quality of work but also prematurely leaving the profession [30,83,84]. Given that teachers’ low PAL is significantly related to their moderately to extremely severe levels of stress, depression, and anxiety, we suggest conducting activities to increase LTPA. Promotion is needed based on awareness of the benefits of PA for health (including mental health) and the introduction of specific LTPA programs (e.g., classes taught by school PE teachers). A supportive leadership style and higher-education management using effective communication, formulating achievable goals, and creating a positive working environment is of great importance in this regard. It is essential to synchronize PA activities with the structural and organizational activities of the respective school and the situation and capabilities of the teacher [85].

## 6. Strengths and Limitations

This is the first study in Poland to analyze simultaneous levels of depression, anxiety, and stress among teachers and the relationship between these disorders and sociodemographic, psychosocial, work, and lifestyle factors of the respondents. The standardized survey instruments DASS-21 and IPAQ-SF were used. Adequate strength of conclusions was guaranteed by adopting an AUC ≥ 0.70, which does not overestimate the strength of the association between variables.

Since this was a small-scale study, among high school teachers in a city within southern Poland, there are several limitations. First, the results cannot be generalized to the entire teaching community. To obtain more accurate data, a more extensive survey with a larger sample is recommended. Second, the DASS-21 is not a diagnostic tool, but a screening tool to detect the presence of symptoms of depression, anxiety, and stress. In a clinical setting, a high DASS-21 score requires evaluation by a psychiatrist before definitive diagnosis and treatment. Therefore, future studies using validated diagnostic tools are recommended to determine the prevalence rates of depression, anxiety, and stress in this community. Finally, the IPAQ was used to estimate the level of PA, which, on the one hand, has some limitations (subjectivity, the effect of the burden of social expectations, or the varying ability of respondents to report past events), but on the other hand, offers the possibility of undertaking a population-based study.

## Figures and Tables

**Table 1:** Overview of analyzed variables in the model with their statistics.

Variables	Operationalization	f	rf (%)
Dependent variable			
**Depression**			
Normal or mild	≤13 pts.	95	81.9
Moderate to extremely severe	≥14 pts.	21	18.1
**Anxiety**			
Normal or mild	≤9 pts.	90	77.6
Moderate to extremely severe	≥10 pts.	26	22.4
**Stress**			
Normal or mild	≤18 pts.	56	48.3
Moderate to extremely severe	≥19 pts.	60	51.7
Independent variable			
**Physical activity level (PAL)**			
High or moderate	≥600 MET-min/week (combined activities of VPA, MPA and walking)	78	67.2
Low	<600 MET-min/week (combined activities of VPA, MPA and walking)	38	32.8
**Gender**			
Male		28	24.1
Female		88	75.9
**Work experience**			
≤20 years		69	59.5
>20 years		47	40.5
**Total lessons per week**			
≤18 h/week		20	17.2
>18 h/week		96	82.8
**Steady income**			
≤2700 PLN (≤€596)		38	32.8
>2700 PLN (>€596)		78	67.2

Notes: f = frequency; rf = relative frequency (percent).

**Table 2:** Recommended cut-off scores for conventional severity labels in DASS-21.

Level	Depression	Anxiety	Stress	Total *
Normal	0–9	0–7	0–14	0–78
Mild	10–13	8–9	15–18	78–87
Moderate	14–20	10–14	19–25	87–95
Severe	21–27	15–19	26–33	95–98
Extremely severe	≥28	≥20	≥34	98–100

Notes: * Scores on the DASS-21 need to be multiplied by 2 in each subscale to calculate the total score.

**Table 3:** Characteristics of mental state and physical activity of general secondary school teachers (*n* = 116).

Variables	f	M	SD	Me	Q1	Q3
**Mental state**						
DS (pts.)	116	7.7	6.8	6.0	2.5	10.0
AS (pts.)	116	6.5	6.2	4.0	2.0	8.0
SS (pts.)	116	19.3	7.4	20.0	14.0	24.0
**Physical activity**						
FVPA (day/week)	40	3.1	1.9	2.0	2.0	4.0
TVPA (min/day)	40	25.9	32.9	10.0	10.0	30.0
EEVPA (MET-min/week)	40	602.0	793.2	320.0	160.0	560.0
FMPA (day/week)	83	3.0	1.9	3.0	2.0	4.0
TMPA (min/day)	83	42.6	32.5	30.0	20.0	60.0
EEMPA (MET-min/week)	83	502.2	518.2	360.0	180.0	600.0
FLPA (day/week)	112	5.9	1.7	7.0	5.0	7.0
TLPA (min/day)	112	33.8	23.2	30.0	20.0	40.0
EELPA (MET-min/week)	112	662.7	516.2	462.0	268.1	693.0
EETPA (MET-min/week)	113	1238.7	976.4	1053.0	542.0	1750.5

Notes: DS = depression scale; AS = anxiety scale; SS = stress scale; FVPA = frequency of vigorous physical activity; TMPA = time of vigorous physical activity; EEVPA = energy expenditure of vigorous physical activity; FMPA = frequency of moderate physical activity; TMPA = time of moderate physical activity; EEMPA = energy expenditure of moderate physical activity; FLPA = frequency of light physical activity; TLPA = time of light physical activity; EELPA = energy expenditure of light physical activity; EETPA = energy expenditure of total physical activity; f = frequency; M = mean; SD = standard deviation; Me = median; Q1 = first quartile, Q3 = third quartile.

**Table 4:** Modeling the probability of normal or mild vs. moderate, severe, or extremely severe symptoms of depression among high school teachers (*n* = 116).

Predictor	Model_1_	Model_2_	Model_3_	Model_4_	Model_5_
	OR (±95% CI)	OR (±95% CI)	OR (±95% CI)	OR (±95% CI)	OR (±95% CI)
**Physical activity level**	AUC = 0.65	AUC = 0.66	AUC = 0.69	AUC = 0.70	AUC = 0.70
High or moderate–low	3.54 * (1.34–9.38)	3.73 * (1.37–10.16)	3.54 * (1.29–9.75)	3.61 * (1.30–10.00)	3.62 * (1.31–10.03)
**Gender**					
Female–male		1.33 (0.41–4.30)	1.37 (0.42–4.44)	1.39 (0.43–4.53)	1.43 (0.43–4.70)
**Work experience**					
≤20 years–>20 years			1.59 (0.59–4.25)	1.57 (0.58–4.20)	1.66 (0.60–4.58)
**Total lessons per week**					
≤18 h/week–>18 h/week				1.38 (0.34–5.54)	1.46 (0.36–6.02)
**Steady income**					
≤2700 PLN–>2700 PLN (≤€596–>€596)					0.77 (0.26–2.30)

Notes: OR = odds ratio; CI = confidence interval for OR; * *p* < 0.05.

**Table 5:** Modeling the probability of normal or mild vs. moderate, severe, or extremely severe symptoms of anxiety among high school teachers (*n* = 116).

Predictor	Model_1_	Model_2_	Model_3_	Model_4_	Model_5_
	OR (±95% CI)	OR (±95% CI)	OR (±95% CI)	OR (±95% CI)	OR (±95% CI)
**Physical activity level**	AUC = 0.61	AUC = 0.62	AUC = 0.65	AUC = 0.67	AUC = 0.68
High or moderate–low	2.60 * (1.06–6.37)	2.42 (0.97–6.01)	2.52 (1.00–6.34)	2.60 * (1.02–6.63)	2.61 * (1.01–6.73)
**Gender**					
Female–male		0.60 (0.18–1.96)	0.59 (0.18–1.94)	0.61 (0.18–2.03)	0.56 (0.17–1.89)
**Work experience**					
≤20 years–>20 years			0.76 (0.30–1.93)	0.73 (0.28–1.85)	0.66 (0.25–1.72)
**Total lessons per week**					
≤18 h/week–>18 h/week				3.22 (0.68–15.22)	2.74 (0.56–13.30)
**Steady income**					
≤2700 PLN–>2700 PLN (≤€596–>€596)					1.82 (0.62–5.38)

Notes: OR = odds ratio; CI = confidence interval for OR; * *p* < 0.05.

**Table 6:** Modeling the likelihood of normal or mild vs. moderate, severe, or extremely severe symptoms of stress among high school teachers (*n* = 116).

Predictor	Model_1_	Model_2_	Model_3_	Model_4_	Model_5_
	OR (±95% CI)	OR (±95% CI)	OR (±95% CI)	OR (±95% CI)	OR (±95% CI)
**Physical activity level**	AUC = 0.61	AUC = 0.65	AUC = 0.66	AUC = 0.67	AUC = 0.70
High or moderate–low	2.74 * (1.21–6.21)	2.46 * (1.07–5.66)	2.61 * (1.12–6.10)	2.71 * (1.15–6.42)	2.79 * (1.16–6.69)
**Gender**					
Female–male		0.42 (0.17–1.05)	0.41 (0.16–1.04)	0.40 (0.16–1.02)	0.35 (0.14–0.92)
**Work experience**					
≤20 years–>20 years			0.67 (0.30–1.48)	0.64 (0.29–1.43)	0.53 (0.23–1.24)
**Total lessons per week**					
≤18 h/week–>18 h/week				1.90 (0.67–5.38)	1.58 (0.54–4.63)
**Steady income**					
≤2700 PLN–>2700 PLN (≤€596–>€596)					2.15 (0.88–5.27)

Notes: OR = odds ratio; CI = confidence interval for OR; * *p* < 0.05.

## Data Availability

The data presented in this study are available on request from the corresponding author.
